# Hypotension prediction index guided versus conventional goal directed therapy to reduce intraoperative hypotension during thoracic surgery: a randomized trial

**DOI:** 10.1186/s12871-023-02069-1

**Published:** 2023-03-30

**Authors:** Andrej Šribar, Irena Sokolović Jurinjak, Hani Almahariq, Ivan Bandić, Jelena Matošević, Josip Pejić, Jasminka Peršec

**Affiliations:** 1 Clinical Department of Anesthesiology, Reanimatology and Intensive Care Medicine, University Hospital Dubrava, Avenija Gojka Šuška 6, 10000 Zagreb, Croatia; 2 Zagreb University School of Dental Medicine, Gundulićeva 5, Zagreb, Croatia; 3 Department of Thoracic Surgery, University Hospital Dubrava, Av. Gojka Šuška 6, Zagreb, Croatia

**Keywords:** Hemodynamic monitoring, Anesthesiology, Machine learning, Intraoperative hypotension, Thoracic surgery

## Abstract

**Purpose:**

Intraoperative hypotension is linked to increased incidence of perioperative adverse events such as myocardial and cerebrovascular infarction and acute kidney injury. Hypotension prediction index (HPI) is a novel machine learning guided algorithm which can predict hypotensive events using high fidelity analysis of pulse-wave contour. Goal of this trial is to determine whether use of HPI can reduce the number and duration of hypotensive events in patients undergoing major thoracic procedures.

**Methods:**

Thirty four patients undergoing esophageal or lung resection were randomized into 2 groups -“machine learning algorithm” (AcumenIQ) and “conventional pulse contour analysis” (Flotrac). Analyzed variables were occurrence, severity and duration of hypotensive events (defined as a period of at least one minute of MAP below 65 mmHg), hemodynamic parameters at 9 different timepoints interesting from a hemodynamics viewpoint and laboratory (serum lactate levels, arterial blood gas) and clinical outcomes (duration of mechanical ventilation, ICU and hospital stay, occurrence of adverse events and in-hospital and 28-day mortality).

**Results:**

Patients in the AcumenIQ group had significantly lower area below the hypotensive threshold (AUT, 2 vs 16.7 mmHg x minutes) and time-weighted AUT (TWA, 0.01 vs 0.08 mmHg). Also, there were less patients with hypotensive events and cumulative duration of hypotension in the AcumenIQ group. No significant difference between groups was found in terms of laboratory and clinical outcomes.

**Conclusions:**

Hemodynamic optimization guided by machine learning algorithm leads to a significant decrease in number and duration of hypotensive events compared to traditional goal directed therapy using pulse-contour analysis hemodynamic monitoring in patients undergoing major thoracic procedures. Further, larger studies are needed to determine true clinical utility of HPI guided hemodynamic monitoring.

**Trial registration:**

Date of first registration: 14/11/2022

Registration number: 04729481-3a96-4763-a9d5-23fc45fb722d

## Introduction

In Europe, approximately 20 million major surgical procedures are performed annually. Up to 5% of these patients will die and 10–15% will develop adverse events that could have been prevented in more than 33% of cases [1]. Reduction of surgical morbidity and mortality is possible by avoiding these events.

Intraoperative hypotension (IOH) may increase mortality and morbidity rates in the postoperative period, and is associated with adverse events such as acute kidney injury, cerebral and myocardial infarction [2]. It is surprisingly common and, depending on its definition, has an incidence range of 5 to 99% [3].

A recent randomized controlled trial reported that preventing intraoperative hypotension reduces the risk of postoperative organ dysfunction by about a quarter [4], while aiming higher MAP values is not strictly necessary in order to reduce the number of perioperative adverse events [5]. It is possible that the ability to recognize when a patient is likely to become hypotensive, as well as the pathophysiology of these events, improves hemodynamic optimization and, potentially, patient outcome.

Absolute threshold in defining IOH includes systolic blood pressure (SBP) less than 90 mmHg or mean arterial pressure (MAP) less than 65 mmHg, while a 20% reduction in baseline SBP or a 30% reduction in MAP represents the relative threshold. Most patients in noncardiac surgery experience at least one episode during which MAP decreases to < 65 mmHg, and known causes are anesthetic drugs, uncorrected hypovolemia, preexisting comorbidities, and surgical manipulation [6, 7].

The “Hypotension Prediction Index” (HPI) is a novel hemodynamic monitoring tool that predicts episodes of intraoperative hypotension before they occur. It has recently been shown that applying Acumen (Edwards Lifesciences, Irvine, Ca, USA) HPI software algorithm on data obtained by invasive blood pressure monitoring sensor during noncardiac surgery enables earlier assessment of causes of impending hypotension and timely response to possible hemodynamic instability [8]. It can predict arterial hypotension with a sensitivity of 88% and specificity of 87% 15 min before; 89% and 90% 10 min before and 92% and 92% 5 min before the occurrence of a hypotensive event (defined as an episode of hypotension < 65 mmHg of at least 1 min) [9].

Given the significant association between hypotension and postoperative complications and adverse outcomes such as increased incidence of myocardial infarction and cerebrovascular insult, as well as increased hospital stay [10], it seems likely that the damage can be mitigated by timely and appropriate anesthesiologist intervention to reduce the occurrence and duration of intraoperative hypotension.

The aim of this study was to examine whether use of machine-learning algorithm guided intraoperative patient hemodynamic optimization reduces duration and severity of hypotension and its complications during and after thoracic surgery compared to conventional pulse contour analysis goal-directed hemodynamic optimization.

## Patients, materials and methods

### Participants and group allocation

By design this study is a prospective, randomized, single blinded study.

Participants are patients over 18 years of age which were scheduled for elective major thoracic procedure (lung resection, pleurectomy or resection of the esophagus) with planned thoracotomy and intraoperative period of one lung ventilation with planned postoperative admission to the ICU. Exclusion criteria were persistent atrial fibrillation, structural heart defects (shunting or moderate to severe valvular anomalies), preoperative serum hemoglobin levels < 120 g/L and severe heart failure classified as New York Heart Association (NYHA) grade IV.

In the anesthesia preparation room, after informed consent was obtained, patients were randomized using coin toss into two groups:“machine learning algorithm”* (AcumenIQ)* group or “conventional pulse contour analysis” (*Flotrac)* group. Both groups were hemodynamically monitored using *Hemosphere* monitoring platform using either *AcumenIQ* or *Flotrac* sensors (Edwards Lifesciences, Irvine, Ca, USA), depending on group allocation.

### Study protocol

#### Hemodynamic measurements

In the operating room and before induction of anesthesia, arterial and central venous lines were placed after sterile skin preparation and local anesthetic infiltration, zeroed to account for hydrostatic pressure differences between the pressure transducer and the heart, and monitoring was initiated.

Following hemodynamic parameters were measured: cardiac index (CI, l/min/m^2^), stroke volume index (SVI, ml/beat/m^2^), systolic, diastolic and mean arterial pressure (mmHg), heart rate (HR, beats per minute), systemic vascular resistance index (SVRI, dyn·s·cm^−5^/m^2^), stroke volume variation (SVV, %), hypotension prediction index (HPI, % - *AcumenIQ* only), systolic pressure increase divided by duration of systole as an indicator of left ventricular contractility (dP/dt, mmHg/sec - *AcumenIQ* only) and dynamic arterial elastance (PPV/SVV, Ea_*dyn*_, expressed in arbitrary units—*AcumenIQ* only).

Hemodynamic measurements were recorded at following time points: at baseline (T0), during induction of anesthesia - 3 min after administration of muscle relaxant (T1), 1 min after intubation (T2), 1 min after placing the patient in lateral decubitus position (T3), 1 min after skin incision (T4), 1 min after thoracotomy and initiation of one-lung ventilation (T5), 1 min after removal of tumor (T6), 3 min after skin closure (T7) and 1 min after placing the patient back in the supine position (T8).

#### Anesthesia protocol and hemodynamics related interventions

Induction of anesthesia was performed using propofol (1–1.5 mg/kg), sufentanil (0.1–0.5 mcg/kg) and rocuronium bromide (0.6 mg/kg). After intubation, placement of Robert Shaw tube was verified capnographically and the correct position of endobronchial lumen was verified using a flexible fiber bronchoscope. Anesthesia was maintained using oxygen/air/sevoflurane mixture at minimal alveolar concentration (MAC) 0.8–1 and FiO_2_ adjusted to achieve peripheral oxygen saturation > 95% during two lung ventilation and > 90% during one lung ventilation. Muscle paralysis was preserved by administration of 8 mcg/kg/min rocuronium bromide.

In patients with thoracic epidural catheter in place, continuous infusion of 1 mg/ml levobupivacaine and 1 mcg/ml sufentanil was administered at rate of 1–2 ml/h, according to attending anesthesiologist’s assessment of patient’s nociceptive response, and in those without an epidural catheter, 5—10 mcg sufentanil i.v. boluses were administered as needed.

Patients were ventilated (Draeger Perseus, Draeger Medical AG, Lübeck, Germany) using a tidal volume of 8 ml/kg_PBW_ during two-lung ventilation and 4–6 ml/kg_PBW_ during one lung ventilation, with frequency set to maintain etCO_2_ 3.5 - 4.5 kPa and I:E ratio was set to allow expiration duration of at least 3 RC.

In the *AcumenIQ* group HPI values ≥ 90 triggered an alert after which the attending anesthesiologist analyzed the *“clinical decision tree”* screen with hemodynamic variables displayed in a structured manner (preload—stroke volume variation, afterload - dynamic arterial elastance and myocardial contractility - rate of systolic pressure change) after which a goal directed therapeutic intervention was performed (i.v volume expansion, vasopressors or inotropes).

In the *Flotrac* group, hemodynamic optimization was guided by measured values displayed on the monitor and interventions were aiming for hemodynamic targets: MAP > 65 mmHg, CI ≥ 2.4 l/min/m^2^, SVI ≥ 30 ml/beat/m^2^ and SVRI 1700–2400 dyn·s·cm^−5^/m^2^.

In supine patients SVV ≥ 13% defined volume responsiveness [11], while it was defined as an SVI increase ≥ 10% after i.v. administration of 4 ml/kg crystalloid solution in patients receiving OLV in lateral decubitus position [12]. In volume responsive patients intravenous crystalloids or colloids were administered until the patient stopped being volume responsive. If CI ≥ 2.4 l/min/m^2^ was not met after adequate volume resuscitation dobutamine infusion was started until the threshold was reached, and if MAP > 65 mmHg was not achieved with adequate CI, norepinephrine was used as a predefined pressor of choice and was administered either as 2–10 mcg bolus or in a continuous infusion until target was achieved.

#### Endpoints

Primary endpoint is time weighted average of area spent under 65 mmHg for MAP per patient (AUT - depth of hypotension below a MAP of 65 mmHg × time spent below a MAP of 65 mm Hg divided by procedure duration in minutes) of AUT (TWA-AUT).

Secondary endpoints are AUT, number of hypotensive episodes (defined as MAP < 65 mmHg for at least 1 min) during the procedure and cumulative duration of hypotension during the procedure.

Volume of i.v. fluids (ml/kg/h) and norepinephrine (mcg/kg/min) administered in the operating theater, urine output (ml/kg/h), postoperative arterial blood pH, arterio-venous CO_2_ gap (kPa) [13], base excess, central venous saturation (scvO_2_, %) and serum lactate levels (mmol/L); duration of postoperative mechanical ventilation (hours), ICU stay (hours), hospital stay (days from day of surgery to discharge) and in-hospital and 28-day mortality, as well as occurrence of postoperative complications: acute kidney injury (defined according to RIFLE criteria [14]), acute coronary syndrome or cerebrovascular infarction during hospital stay are also reported.

### Data acquisition and statistical analysis

Hemodynamic measurements for each patient were exported from *Hemosphere* monitor via USB as Microsoft Excel (Microsoft Corporation, Redmond, Wa, USA).xls files and were analyzed with the *Acumen Analytics* (Edwards Lifesciences, Irvine, Ca, USA) software package which generated reports from which derived data about intraoperative hypotension events was collected.

Continuous variables are displayed as either mean and standard deviation (SD) for values with Gaussian distribution, or median and interquartile range or 95% confidence interval (CI) for data that does not follow normal distribution. Normality of distribution was assessed using the Shapiro–Wilk test. Categorical variables are displayed as counts and percentages.

Differences in independent continuous variables between 2 groups were tested for statistical significance using Student’s t test for independent samples or Mann–Whitney U test, depending on distribution of data. For measurements at various time points, repeated measurements two-way analysis of variance (RM-ANOVA) with post-hoc Holm-Bonferroni correction was performed, and values are expressed as marginal mean and 95% confidence interval (CI). Differences between groups in categorical variables were tested for statistical significance using χ^2^ or Fisher’s exact test for 2 × 2 tables.

Pilot trial with 5 participants per group was conducted for difference in means of TWA-AUT and effect size (d) of 1.08 was obtained. For ɑ error probability of 0.05 and statistical power (1-ꞵ) of 0.8 sample size of minimally 16 patients per group was calculated. Software package G*Power v 3.1.9.7 [15, 16] was used for sample size calculation and participants from the pilot analysis were not included in the final analysis.

*P* values < 0.05 were considered statistically significant. Software package jamovi v2.3.2 [17] was used for statistical analysis and data visualization.

## Results

There were 36 patients screened for inclusion, of which 1 was not randomized due to presence of an exclusion criterion, and 1 declined to participate. 34 participants were included, of which 17 were randomized to be monitored with the *AcumenIQ* sensor which uses machine learning guided HPI algorithm, and 17 were monitored with the *Flotrac* sensor and conventional goal directed therapy was utilized.

No statistically significant differences between groups were observed in regard to their baseline demographic, clinical and anthropometric data, severity of comorbidities (Charlson comorbidity index—CCI) as well as type of surgery performed, and most hemodynamic measurements. Only baseline heart rate was higher in the AcumenIQ group (Table 1).

**Table 1:** Baseline patient data

	**AcumenIQ**	**Flotrac**	**Test Statistic**
	(*N* = 17)	(*N* = 17)	
**Sex (F/M)**	**7/10**	**9/8**	Χ^2^ = 0.47, *P* = 0.49^2^
**Age (y)**	59.7 **65.0** 67.0	61.7 **69.0** 71.3	*F* = 1.31, *P* = 0.26^3^
**Height (cm)**	165.0 **168.0** 173.7	158.3 **170.0** 178.7	*F* = 0.00, *P* = 0.97^3^
**Weight (kg)**	74.3 **86.0** 95.7	66.3 **77.0** 86.0	*F* = 3.10, *P* = 0.09^3^
**BMI (kg/m**^**2**^**)**	25.3 **31.0** 33.1	23.1 **26.2** 30.6	*F* = 3.09, *P* = 0.09^3^
**CCI**	4.0 **4.0** 5.3	4.0 **5.0** 6.3	*F* = 3.41, *P* = 0.07^3^
**ASA**	2.0 **3.0** 3.0	2.0 **3.0** 3.0	*F* = 0.42, *P* = 0.52^3^
**SVI (ml/beat/m**^**2**^**)**	32.0 **35.0** 41.3	32.8 **38.5** 45.6	*F* = 0.71, *P* = 0.41^3^
**CI (l/min)**	2.5 **2.9** 3.3	2.4 **2.7** 3.1	*F* = 0.62, *P* = 0.44^3^
**HR (bpm)**	70.3 **82.0** 92.0	60.7 **74.0** 77.7	***F* = 4.68, *P* = 0.04**^**3**^
**Systolic BP (mmHg)**	144.7 **157.0** 173.7	145.3 **156.0** 176.0	*F* = 0.05, *P* = 0.83^3^
**Diastolic BP (mmHg)**	71.7 **81.0** 88.3	67.7 **86.0** 90.7	*F* = 0.09, *P* = 0.76^3^
**MAP (mmHg)**	97.3 **112.0** 120.3	94.3 **112.0** 125.7	*F* = 0.15, *P* = 0.70^3^
**SVRI (dyn·s·cm**^**−5**^**/m**^**2**^**)**	2153.5 **2429.0** 3302.7	2087.5 **2731.0** 3718.3	*F* = 0.00, *P* = 0.97^3^
**Op: Lung / Esophagus**	**14/3**	**14/3**	Χ^2^ = 0.00, *P* = 1.00^2^
**Analgesia: epidural / i.v**	**13/4**	**11/6**	Χ^2^ = 0.57, *P* = 0.45^2^
**Procedure duration (min)**	123.3 **165.0** 228.3	150.0 **180.0** 185.0	*F* = 0.08, *P* = 0.77^3^

Median and IQR. ^1^Kruskal-Wallis, ^2^chi-squared, ^3^Mann-Whitney

**Fig. 1:**
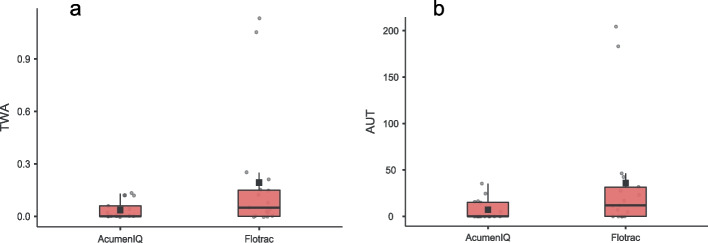
**a** area under 65 mmHg (mmHg x minutes); **b** TWA of AUT (mmHg)

In the *Flotrac* group there were a total of 50 hypotensive events (13/17 patients) with a total duration of hypotension of 105 min, compared to 13 events in the *AcumenIQ* group (7/17 patients) with total duration of hypotension of 22 min. Patients in the *AcumenIQ* group had significantly lower number of hypotensive events, duration of hypotensive event per patient and area under 65 mmHg for MAP per patient (AUT) and weighted average (TWA) of AUT. There was no significant difference in the number of patients that were hypotensive under 50 mmHg and MAP during hypotensive episodes (Fig. 1a and b*, *Table 2).

**Table 2:** Primary and secondary outcomes

	**AcumenIQ**	**Flotrac**	**Test Statistic**
	**(*N* = 17)**	**(*N* = 17)**	
**Number of hypotensive pts**	7/17	13/17	**Χ**^**2**^** = 4.37, *P* = 0.04**^**2**^
**Hypotensive events per patient**	0.0 **0.0** 1.3	0.7 **2.0** 3.3	***F* = 6.94, *P* = 0.01**^**3**^
**Duration of hypotension (min)**	0.0 **0.0** 2.2	0.7 **3.7** 7.8	***F* = 7.61, *P* = 0.01**^**3**^
**MAP during event (mmHg)**	60.6 **62** 63.3	60 **61.2** 62.2	*F* = 0.02, *P* = 0.90^3^
**AUT (mmHg x minutes)**	0.6 **2.0** 15.2	3.6 **16.7** 43.3	***F* = 4.41, *P* = 0.04**^**3**^
**TWA of AUT (mmHg)**	0 **0.01** 0.08	0.02 **0.08** 0.22	***F* = 4.33, *P* = 0.04**^**3**^
**Patients with MAP < 50 mmHg**	0/17	2/17	Χ^2^ = 2.13, *P* = 0.15^2^
**Crystalloids (ml/kg/h)**	9.5 **11.8** 14.5	11.1 **13.6** 16.2	*F* = 0.89, *P* = 0.35^3^
**Colloids (ml/kg/h)**	0.0 **0.0** 1.5	0.0 **0.0** 2.3	*F* = 0.14, *P* = 0.72^3^
**RBC (ml/kg/h)**	0.0 **0.0** 0.2	0.0 **0.0** 0.0	*F* = 2.47, *P* = 0.13^3^
**Norepinephrine (µg/kg/min)**	.01 **.01** .02	.00 **.01** .03	*F* = 0.09, *P* = 0.76^3^
**Urine output (ml/kg/h)**	2.3 **2.7** 3.6	2.6 **3.4** 5.1	*F* = 1.26, *P* = 0.27^3^
**Lactate (mmol/L)**	0.7 **0.9** 1.2	0.7 **1.1** 1.3	*F* = 0.68, *P* = 0.42^3^
**BE (mmol/L)**	-2.8 **-1.0** 0.8	-3.8 -**1.7** -0.8	*F* = 1.61, *P* = 0.21^3^
**pH**	7.3 **7.3** 7.4	7.3 **7.3** 7.4	*F* = 1.01, *P* = 0.32^3^
**ScVO**_**2**_** (%)**	74.0 **81.2** 84.1	71.0 **74.8** 81.7	*F* = 1.22, *P* = 0.28^3^
**Arterio-venous CO**_**2**_** gap (kPa)**	0.7 **0.9** 1.1	0.5 **0.9** 1.2	*F* = 0.14, *P* = 0.71^3^
**Mechanical ventilation (h)**	0.0 **0.0** 2.2	0.0 **0.0** 0.0	*F* = 1.51, *P* = 0.23^3^
**ICU stay (h)**	19.0 **21.5** 28.7	19.7 **22.0** 22.0	*F* = 0.09, *P* = 0.77^3^
**Hospital stay (d)**	7.0 **9.0** 12.3	7.0 **7.0** 9.3	*F* = 2.23, *P* = 0.15^3^
**Myocardial infarction (Y/N)**	0/17	0/17	N/A
**Cerebrovascular infarction (Y/N)**	0/17	0/17	N/A
**Acute kidney injury (Y/N)**	0/17	1/16	Χ^2^ = 1.03, *P* = 0.31^2^
**Alive/dead at discharge**	16/1	17/0	Χ^2^ = 1.03, *P* = 0.31^2^
**Alive/dead at day 28**	16/1	17/0	Χ^2^ = 1.03, *P* = 0.31^2^

Median and IQR. ^1^Kruskal-Wallis, ^2^chi-squared, ^3^Mann-Whitney

There was no significant difference between groups in the amount of administered intraoperative i.v. fluids, packed red blood cells or norepinephrine, clinical (urine output, duration of postoperative mechanical ventilation, duration of ICU and hospital stay, hospital and 28 day mortality, incidence of myocardial infarction, cerebrovascular infarction or acute kidney injury during hospital stay) or laboratory (pH, BE, CO_2_ gap, lactate, ScvO_2_) outcomes (Table 2*).*

**Fig. 2:**
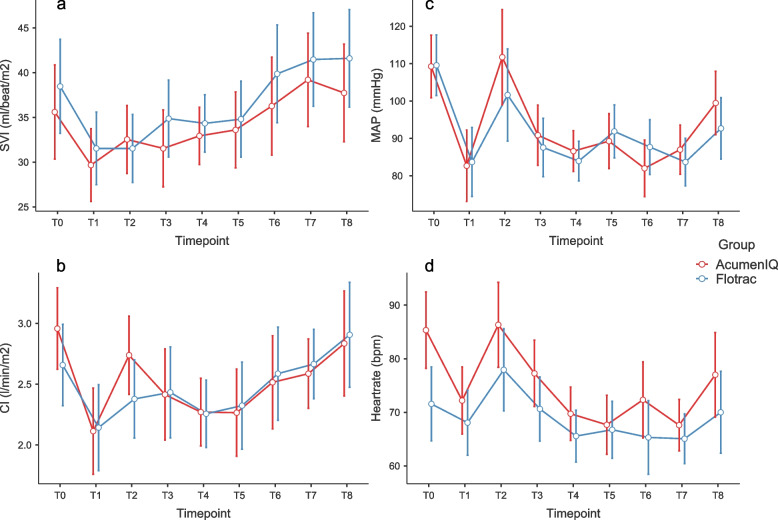
Changes of SVI (**a**), CI (**b**), MAP (**c**) and HR (**d**) over measurements, marginal means and 95% CI error bars

In terms of changes of hemodynamic parameters at timepoints, after post-hoc correction for multiple measurements no statistically significant difference was measured between groups and measurements for SVI (*P* = 0.915, _P_η^2^ = 0.014—Fig. 2a), while there was a significant within-group drop in CI during induction (*P* = 0.002) and an increase after intubation in the *AcumenIQ* group (*P* < 0.001), but without difference between groups (*P* = 0.490, _P_η^2^ = 0.032—Fig. 2b). There was a significant within-group drop in MAP during induction in both groups (*Flotrac* *P* < 0.001, *AcumenIQ* *P* < 0.001) and an increase after intubation in the *AcumenIQ* group (*P* = 0.002), but without difference between groups (*P* = 0.518, _P_η^2^ = 0.03—Fig. 2c).

**Fig. 3:**
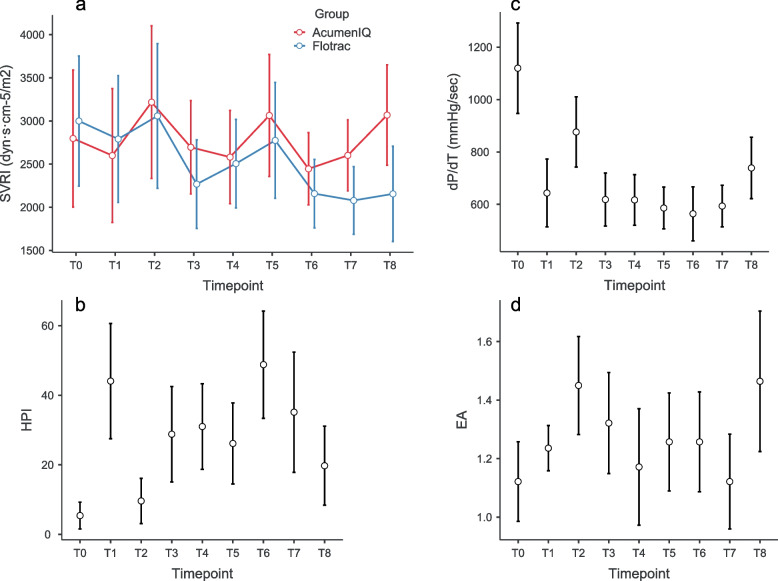
Changes of SVRI (**a**), HPI (**b**), dT/dT (**c**) and EA_dyn_ (**d**) over measurements, marginal means and 95% CI error bars

A significant heart rate increase in the AcumenIQ group was observed after intubation (*P* = 0.014) but without other significant differences between groups and measurements (*P* = 0.174, _P_η^2^ = 0.048—Fig. 2d). No significant differences in SVRI (*P* = 0.226, _P_η^2^ = 0.073—Fig. 3a) were observed between groups and measurements.

Regarding changes of parameters that could only be measured with the *AcumenIQ* sensor, there was a significant increase of hypotension prediction index during anesthesia induction (*P* = 0.004) and drop after intubation (*P* = 0.006; _P_η^2^ = 0.315—Fig. 3b), and a significant drop of myocardial contractility (dP/dT) during induction (*P* < 0.001), followed by an increase after intubation (*P* = 0.001; _P_η^2^ = 0.526—Fig. 3c). No significant change of dynamic arterial elastance was observed between adjacent measurements (*P* = 0.011 before post-hoc correction; _P_η^2^ = 0.170—Fig. 3d).

## Discussion

In the studied cohort, use of machine learning algorithm guided hemodynamic optimization (HPI) significantly reduced the number and duration of intraoperative hypotensive episodes, as well as AUT and the primary outcome—TWA of AUT.

These results are in agreement with results obtained by Wijnberge et al. [8] and Grundmann et al. [18] while results published by Maheshwari et al. [19] show that intraoperative use of machine learning guided prediction of hypotension did not reduce the number and duration of hypotensive episodes. When comparing the results of these studies, it must be noted that aforementioned studies included fairly heterogeneous subjects, with different types of surgical procedures performed, while patients in our cohort were a homogenous population from a surgical intervention standpoint (performed procedures were either lung or esophagus resection), which eliminated the confounding effect of different surgical procedures on hemodynamic stability. Also, post-hoc analysis of a subset of patients in trial by Maheshwari et al. showed that use of hypotension prediction index guidance was associated with less hypotension when analysis was restricted to episodes during which clinicians intervened after hypotension prediction warning alarm—once again showing that any monitoring method is only as good as the clinician interpreting the measured data and reacting upon it is.

A recent study by Enevoldsen et al. [20] discussed a problematic selection bias in the development of the HPI algorithm which might overestimate the probability of occurrence of a hypotensive event after HPI levels have reached a certain threshold (provided no therapeutic intervention was performed after the alarm). While these concerns are valid from a statistical point of view, the goal of HPI guided hemodynamic monitoring is to reduce the incidence and duration of hypotensive events, which was achieved both in our study and study by Wijnberge et al.

Our results show that while incidence and duration of hypotension were significantly reduced in patients that used HPI algorithm, there were no significant differences in clinical outcomes, however these results should be interpreted with caution due to sample size and inadequate statistical power. Results of a large retrospective study by Gregory et al. [10] (over 350 000 patients) suggest that every 5 mmHg decrease in MAP under 65 mmHg is associated with an 1.17 OR of adverse cardiac or cerebrovascular events. In our cohort, median MAP during hypotensive events was 62 and 61.2 mmHg respectively, therefore MAP decrease did not reach a 5 mmHg decrease threshold. Also, odds ratios for myocardial infarction and acute kidney injury for cumulative time (by 5-min increments) under MAP < 65 mmHg were 1.02 (i.e. 2% increase in odds for every 5 min of hypotension) while median duration of hypotension in the *Flotrac* group was 3.7 min and 0.0 min in the *AcumenIQ* group. These results show that both reactive or proactive approaches to hemodynamic optimization will achieve similar clinical outcomes, as long as the anesthesiologist's reaction to occurrence of intraoperative hypotension is rapid and goal directed.

There was one patient in the *AcumenIQ* group (who had zero hypotensive events) that died in the hospital due to surgical complications which required subsequent re-do procedures and resulted in a fatal outcome on 27^th^ post-op day.

While hemodynamic stability was much better preserved in the *AcumenIQ* group, there was no significant difference in the amount of i.v. fluid or vasopressors administered between groups. In a study by Wu et al. [21] both overly restrictive and overly liberal approaches may increase incidence of postoperative pulmonary complications in patients undergoing lung resection, while research published by Li et al. has shown that there is no significant difference between restrictive and goal directed approach at fluid resuscitation regarding outcomes [22]. Patients in our cohort received more i.v. crystalloids and less colloids and norepinephrine compared to patients in that study, with similar outcomes.

Hypotension prediction index has proven to be reliable enough in prediction of intraoperative hypotensive events even when used with non-invasive volume clamp method, with similar sensitivity and specificity levels as when used with arterial lines [23, 24].

Since most elective thoracic procedures (lung and esophagus resection) are generally not associated with major blood loss and fluid shift, most hemodynamic changes are due to surgical manipulation, the patient’s position and changes in cardiac output during OLV, which rises after pleural opening [25]. In our study, there were no significant differences between cardiac or stroke volume index after pleural opening (Fig. 2a and b).

One probable reason why both incidence and duration of hypotensive events were significantly lower in the *AcumenIQ* group is the fact that dynamic parameters such as stroke volume variation are not a viable method to determine fluid responsiveness during OLV, and once hypotension occurs, valuable time is lost during a fluid responsiveness trial, while once the HPI alarm goes off, there is enough time to react before the hypotension itself and it can be either avoided, or its duration can be reduced, as our results show.

Further extension of machine learning algorithms in order to preserve intraoperative hemodynamic stability is application of closed loop systems that automatically administer fluids and vasopressors according to measured hemodynamic variables, which have reduced duration of intraoperative hypotension (< 65 mmHg) by 21.1%, as well as a reduction of intraoperative fluid balance (+ 1600 vs + 2050 ml) and lower serum lactate levels (1.2 vs 2.7 mmol/L) in patients undergoing moderate to high-risk surgery as demonstrated by Joosten et al. [26].

It must be noted that most patients in both groups were graded as ASA 3, and although significant comorbidities are present (most of which are associated with smoking), careful multidisciplinary approach and preoperative assessment of cardiac and lung reserve are essential in decreasing the duration of mechanical ventilation, ICU and hospital stay, and improving patient outcomes in general [27].

### Study limitations

Since surgical manipulation (such as heart, lung or major vascular structure compression leading to preload drop) in thoracic procedures will result in rapid hemodynamic changes that cannot be predicted by an algorithm, there were concerns that use of HPI to guide hemodynamic optimization in such procedures is futile. However, obtained results show that even with that shortcoming, there were significant differences between groups in hypotension related outcomes.

One other question that is usually raised in studies which rely on arterial waveform pulse contour analysis is their utility in patients with an open thorax and/or reduced left ventricular ejection fraction, as studied by Vetrugno et al. in patients that were monitored using second generation algorithm [28]. In our study, latest, fourth generation pulse contour analysis algorithm was used, and although it still doesn’t reach acceptable agreement with the golden standard (pulmonary arthery catheter measured cardiac output) in extreme scenarios such as in patients with severe aortic stenosis [29] or patients with left-ventricular ejection fraction < 35% [30] in general it is considered an improvement with increased accuracy after vasopressor administration [31], increased accuracy in patients with ectopic beats and a reduction of hospital and ICU length of stay associated with this method of hemodynamic monitoring in patients undergoing coronary artery bypass grafting [32]. It is also important to remember that primary observed outcome was the TWA-AUT which is calculated from blood pressure values, and not from cardiac output, and because of that, accuracy of pulse contour analysis is of lesser importance compared to previously mentioned studies which compared measured cardiac output (and derived) values.

In terms of the number of participants included in this study, sample size was determined for primary outcome measure, for which the null hypothesis was rejected. However, in terms of secondary clinical outcomes, the study is underpowered, and a much larger sample is needed to draw relevant conclusions. For example, while there was no significant difference in lactate levels between groups (0.9 vs 1.1 mmol/L, *P* = 0.42), statistical power is 0.60 and to achieve 0.8 at least 45 participants per group are needed. Due to the fact that no external funding was received and considering the cost of a single-use pressure transducer set needed for hemodynamic monitoring, we were unable to conduct a larger study.

Secondly, we decided not to routinely check for serum troponin levels to determine whether the patient developed myocardial infarction, since elevated serum troponin is common after lung resection (up to 49% patients) [33] but opted for clinical presentation and ECG changes coupled with an increase of serum troponin as a confirmation of myocardial infarction.

Also, during statistical analysis, some concerns were raised about the validity of the coin-toss method of randomization. However, since there were no significant differences in relevant baseline characteristics between groups, choice of randomization method did not result in sampling bias, as can be seen in Table 1.

## Conclusion

Results of this study suggest that use of machine learning algorithm guided hemodynamic optimization can significantly decrease both number and duration of hypotensive events compared to conventional pulse contour analysis guided monitoring in patients undergoing major thoracic procedures. There were no significant differences between groups regarding secondary clinical outcomes and larger multi-center studies are needed to determine true clinical utility and feasibility of predictive hemodynamic monitoring in thoracic surgery.

## Data Availability

The datasets generated and/or analyzed during the current study are not publicly available due to authors’ fear that they will be used without consent but are available from the corresponding author on reasonable request after the manuscript has been published.

## References

[1] Dobson GP (2020). Trauma of major surgery: a global problem that is not going away. Int J Surg.

[2] Salmasi V, Maheshwari K, Yang D, Mascha EJ, Singh A, Sessler DI (2017). Relationship between intraoperative hypotension, defined by either reduction from baseline or absolute thresholds, and acute kidney and myocardial injury after noncardiac surgery: a retrospective cohort analysis. Anesthesiology.

[3] Bijker JB, van Klei WA, Kappen TH, van Wolfswinkel L, Moons KGM, Kalkman CJ (2007). Incidence of intraoperative hypotension as a function of the chosen definition: literature definitions applied to a retrospective cohort using automated data collection. Anesthesiology.

[4] Futier E, Lefrant JY, Guinot PG, Godet T, Lorne E, Cuvillon P (2017). Effect of individualized vs standard blood pressure management strategies on postoperative organ dysfunction among high-risk patients undergoing major surgery: a randomized clinical trial. JAMA.

[5] Wanner PM, Wulff DU, Djurdjevic M, Korte W, Schnider TW, Filipovic M (2021). Targeting higher intraoperative blood pressures does not reduce adverse cardiovascular events following noncardiac surgery. J Am Coll Cardiol.

[6] Tritapepe L (2013). Hypotension during surgery for high risk patients: cause or consequence of pathology?. Minerva Anestesiol.

[7] Cheung CC, Martyn A, Campbell N, Frost S, Gilbert K, Michota F (2015). Predictors of intraoperative hypotension and bradycardia. Am J Med.

[8] Wijnberge M, Geerts BF, Hol L, Lemmers N, Mulder MP, Berge P (2020). Effect of a machine learning-derived early warning system for intraoperative hypotension vs standard care on depth and duration of intraoperative hypotension during elective noncardiac surgery: the HYPE randomized clinical trial. JAMA.

[9] Hatib F, Jian Z, Buddi S, Lee C, Settels J, Sibert K (2018). Machine-learning algorithm to predict hypotension based on high-fidelity arterial pressure waveform analysis. Anesthesiology.

[10] Gregory A, Stapelfeldt WH, Khanna AK, Smischney NJ, Boero IJ, Chen Q (2021). Intraoperative hypotension is associated with adverse clinical outcomes after noncardiac surgery. Anesth Analg.

[11] Zlicar M, Novak-Jankovic V, Blagus R, Cecconi M (2018). Predictive values of pulse pressure variation and stroke volume variation for fluid responsiveness in patients with pneumoperitoneum. J Clin Monit Comput.

[12] Messina A, Palandri C, De Rosa S, et al. Pharmacodynamic analysis of a fluid challenge with 4 ml kg−1 over 10 or 20 min: a multicenter cross-over randomized clinical trial. J Clin Monit Comput. 2022;36:1193–203. 10.1007/s10877-021-00756-3. 10.1007/s10877-021-00756-3 PMC8423602 34494204

[13] Scheeren TWL, Wicke JN, Teboul JL (2018). Understanding the carbon dioxide gaps. Curr Opin Crit Care.

[14] Bellomo R, Kellum JA, Ronco C (2007). Defining and classifying acute renal failure: from advocacy to consensus and validation of the RIFLE criteria. Intensive Care Med.

[15] Buchner A, Erdfelder E, Faul F, Lang AG. G*Power. Universität Düsseldorf. Available from: https://www.psychologie.hhu.de/arbeitsgruppen/allgemeine-psychologie-und-arbeitspsychologie/gpower. [Cited 2022 Jul 2].

[16] Faul F, Erdfelder E, Lang AG, Buchner A (2007). G*Power 3: A flexible statistical power analysis program for the social, behavioral, and biomedical sciences. Behav Res Methods.

[17] The jamovi project. jamovi, www.jamovi.org. 2019. Available from: www.jamovi.org.

[18] Grundmann CD, Wischermann JM, Fassbender P, Bischoff P, Frey UH (2021). Hemodynamic monitoring with hypotension prediction index versus arterial waveform analysis alone and incidence of perioperative hypotension. Acta Anaesthesiol Scand.

[19] Maheshwari K, Shimada T, Yang D, Khanna S, Cywinski JB, Irefin SA (2020). Hypotension prediction index for prevention of hypotension during moderate- to high-risk noncardiac surgery. Anesthesiology.

[20] Enevoldsen J, Vistisen ST (2022). Performance of the hypotension prediction index may be overestimated due to selection bias. Anesthesiology.

[21] Wu Y, Yang R, Xu J, Rusidanmu A, Zhang X, Hu J (2019). Effects of intraoperative fluid management on postoperative outcomes after lobectomy. Ann Thorac Surg.

[22] Li M, Peng M (2021). Prospective comparison of the effects of intraoperative goal-directed fluid therapy and restrictive fluid therapy on complications in thoracoscopic lobectomy. J Int Med Res.

[23] Frassanito L, Giuri PP, Vassalli F, Piersanti A, Longo A, Zanfini BA, et al. Hypotension Prediction Index with non-invasive continuous arterial pressure waveforms (ClearSight): clinical performance in Gynaecologic Oncologic Surgery. J Clin Monit Comput. 2021. Available from: 10.1007/s10877-021-00763-4. [Cited 2022 Jun 29]. 10.1007/s10877-021-00763-4 PMC8496438 34618291

[24] Maheshwari K, Buddi S, Jian Z, Settels J, Shimada T, Cohen B (2021). Performance of the hypotension prediction index with non-invasive arterial pressure waveforms in non-cardiac surgical patients. J Clin Monit Comput.

[25] Kittnar O (2011). Cardiac preload: hemodynamic physiology during thoracic surgery. Curr Opin Anaesthesiol.

[26] Joosten A, Rinehart J, Van der Linden P, Alexander B, Penna C, De Montblanc J (2021). Computer-assisted individualized hemodynamic management reduces intraoperative hypotension in intermediate- and high-risk surgery: a randomized controlled trial. Anesthesiology.

[27] Della Rocca G, Vetrugno L, Coccia C, Pierconti F, Badagliacca R, Vizza CD, Papale M, Melis E, Facciolo F (2016). Preoperative evaluation of patients undergoing lung resection surgery: defining the role of the anesthesiologist on a multidisciplinary team. J Cardiothorac Vasc Anesth.

[28] Vetrugno L, Costa MG, Spagnesi L, Pompei L, Chiarandini P, Gimigliano I, Della RG (2011). Uncalibrated arterial pulse cardiac output measurements in patients with moderately abnormal left ventricular function. J Cardiothorac Vasc Anesth.

[29] Eisenried A, Klarwein R, Ihmsen H, Wehrfritz A, Tandler R, Heim C, Fechner J (2019). Accuracy and trending ability of the fourth-generation FloTrac/EV1000 system in patients with severe aortic valve stenosis before and after surgical valve replacement. J Cardiothorac Vasc Anesth.

[30] Hattori K, Maeda T, Masubuchi T, Yoshikawa A, Ebuchi K, Morishima K, Kamei M, Yoshitani K, Ohnishi Y (2017). Accuracy and trending ability of the fourth-generation FloTrac/Vigileo system in patients with low cardiac index. J Cardiothorac Vasc Anesth.

[31] Ji F, Li J, Fleming N, Rose D, Liu H (2015). Reliability of a new 4th generation FloTrac algorithm to track cardiac output changes in patients receiving phenylephrine. J Clin Monit Comput.

[32] Tribuddharat S, Sathitkarnmanee T, Ngamsangsirisup K, Nongnuang K (2021). Efficacy of intraoperative hemodynamic optimization using FloTrac/EV1000 platform for early goal-directed therapy to improve postoperative outcomes in patients undergoing coronary artery bypass graft with cardiopulmonary bypass: a randomized controlled trial. Med Devices (Auckl).

[33] Uchoa RB, Caramelli B (2020). Troponin I as a mortality marker after lung resection surgery – a prospective cohort study. BMC Anesthesiol.

